# Evaluation of Peri-Implant Bone Grafting Around Surface-Porous Dental Implants: An In Vivo Study in a Goat Model

**DOI:** 10.3390/ma12213606

**Published:** 2019-11-03

**Authors:** Fahad Alshehri, Mohammed Alshehri, Terrence Sumague, Abdurahman Niazy, John Jansen, Jeroen van den Beucken, Hamdan Alghamdi

**Affiliations:** 1 Department of Periodontics and Community Dentistry, College of Dentistry, King Saud University, Riyadh 11545, Saudi Arabia; fahalshehri@ksu.edu.sa; 2 Dental Department, King Khalid University Hospital, King Saud University, Riyadh 11545, Saudi Arabia; dr_mzs@hotmail.com; 3 Molecular and Cell Biology Laboratory, College of Dentistry, King Saud University, Riyadh 11545, Saudi Arabia; tsumague@ksu.edu.sa (T.S.); aaniazy@ksu.edu.sa (A.N.); 4 Department of Regenerative Biomaterials, Radboudumc, 6525EX Nijmegen, The Netherlands; John.Jansen@radboudumc.nl (J.J.); Jeroen.vandenBeucken@radboudumc.nl (J.v.d.B.)

**Keywords:** dental implants, porous titanium, biphasic calcium phosphate, bone regeneration

## Abstract

Dental implants with surface-porous designs have been recently developed. Clinically, peri-implant bone grafting is expected to promote early osseointegration and bone ingrowth when applied with surface-porous dental implants in challenging conditions. The aim of this study was to comparatively analyze peri-implant bone healing around solid implants and surface-porous implants with and without peri-implant bone grafting, using biomechanical and histomorphometrical assessment in a goat iliac bone model. A total of 36 implants (4.1 mm wide, 11.5 mm long) divided into three groups, solid titanium implant (STI; *n* = 12), porous titanium implants (PTI; *n* = 12) and PTI with peri-implant bone grafting using biphasic calcium phosphate granules (PTI + BCP; *n* = 12), were placed bilaterally in the iliac crests of six goats. The goats were sacrificed seven weeks post-operatively and then subjected to biomechanical (*n* = 6 per group) and histomorphometrical (*n* = 6 per group) assessment. The biomechanical assessment revealed no significant differences between the three types of implants. Although the peri-implant bone-area (PIBA%) measured by histomorphometry (STI: 8.63 ± 3.93%, PTI: 9.89 ± 3.69%, PTI + BCP: 9.28 ± 2.61%) was similar for the three experimental groups, the percentage of new bone growth area (BGA%) inside the porous implant portion was significantly higher (*p* < 0.05) in the PTI group (10.67 ± 4.61%) compared to the PTI + BCP group (6.50 ± 6.53%). These data demonstrate that peri-implant bone grafting around surface-porous dental implants does not significantly accelerate early osseointegration and bone ingrowth.

## 1. Introduction

Titanium implants, known for their high degree of biocompatibility and good mechanical properties, are the most frequently used endosseous implants in dentistry and orthopedics [1]. In fact, it has been reported that the number of placed dental implants is expected to increase every year due to the need to replace missing teeth [2]. Dental implants offer the advantage of enhanced durability, improved aesthetics, convenience, comfort, and oral hygiene [3]. The success rate of dental implants is affected by the mechanical engagement of the implant with the surrounding bone ensuring primary stability, which is determined by the quantity and the quality of the bone, implant geometry, and surface modifications, and the applied surgical technique [4,5,6,7,8]. Therefore, the long-term survival rate of dental implants is challenged by low-density bone (Type IV bone, Lekholm and Zarb classification) [9], as is frequently seen in elderly patients [10]. This suboptimal type IV bone leads to the clinical instability of dental implants, due to unsatisfactory osseointegration [11]. Thus, the decreased capacity of bone to optimize implant fixation can be considered as a potential risk factor for implant failure [12].

Currently, appropriate biological fixation, initial and long-term stability have been demonstrated by porous surface modification, which aids in bone ingrowth improvements [13,14,15,16]. In recent years, researchers have developed highly surface-porous implants with trabecular bone-like surface topography (Trabecular Metal Zimmer^®^, Dental Implant System, Parsippany, NJ, USA), which are expected to improve biomechanics and the biological properties of dental implants by increasing their surface interactions with bone tissue [5,6,17]. The concept of incorporating porous tantalum trabecular metal (PTTM) into a titanium implant fixture was first used for orthopedic treatment [5]. However, the quality and quantity of bone ingrowth are determined by the numbers and sizes of porosities that can be created on the implant surface [14]. It has been reported that a pore size larger than 150 μm provides a favorable environment for bone formation and growth inside the porous material [15,16]. Indeed, the used of surface-porous implants were often prescribed for immediate implant placement with peri-implant bone grafting.

Peri-implant bone grafting plays a vital role in dental implant therapy when the quality and quantity of the alveolar bone are compromised as in type IV bone [18]. It can be done through various techniques of guided bone regeneration utilizing autografts, allografts, xenografts, and synthetic bone substitutes [19]. Peri-implant bone grafting with synthetic bone substitutes has been clinically pioneered to increase implant anchorage at the time of insertion, especially in conditions of low bone quality and reduced trabeculation [20,21,22,23]. A similar surgical technique has been frequently described in orthopedic treatment, called impaction bone grafting (IBG) [24]. In dentistry, the method of peri-implant bone grafting has encouraged immediate implant placement especially in large tooth-extraction sockets or in the case of maxillary sinus augmentation [25,26]. Currently, calcium phosphate (CaP) based ceramics have seen a wide range of applications among bone substitute materials due to their suitable bioactivity, near similar bone mineral composition, and ability to promote cellular function [27,28]. CaP-based bone grafts are commonly synthesized of either hydroxyapatite (HA), tricalcium phosphate (TCP), or a biphasic combination (TCP/HA) [29]. Biphasic calcium phosphate (BCP) possesses substantial advantages over HA and TCP, due to its osteoinductive ability [30]. Hence, the use of BCP for peri-implant bone grafting around surface-porous dental implants could play a pivotal role to accelerate early osseointegration and bone ingrowth, subsequently improving the implant stability, epically in low-quality and low-quantity bone.

In order to perform an in vivo experiment in challenging bone conditions, the iliac crest in goats has been suggested to resemble type IV bone with compromised trabeculation, as in posterior maxilla [31]. In addition, the iliac crest model allows a bilateral setup, which has a considerable effect in reducing the number of animals and associated costs [32].

Therefore, the aim of the present study was to comparatively analyze peri-implant bone healing around solid implants and surface-porous implants with and without BCP peri-implant bone grafting, using biomechanical and histomorphometrical assessment in a goat iliac bone model.

## 2. Materials and Methods

### 2.1. Animal Model

Six healthy male goats with a mean age of 18 ± 0.9 months (range 17–19 months) and mean weight 36.9 ± 0.45 kg (range 36.4–37.6 kg) were used in the study. The animals were under veterinary supervision at the animal facility. Preoperative acclimatization of the animals to the laboratory environment was ensured for a period of 7 days at a constant temperature of 27 °C and humidity of 60% with 12 hourly nocturnal cycles. The present animal study was conducted in accordance with the NIH guidelines for the care and use of laboratory animals (NIH publication #85-23 Rev. 1985). The work-protocol of the animal experiment was conducted using the facility and support of the College of Dentistry, King Saud University, Riyadh, Saudi Arabia, with the ethical reference number (FR0263).

### 2.2. Study Design

A total of 36 dental implants (4.1 mm diameter and 11.5 mm length) were inserted in the bilateral iliac crests of 6 goats.

The implants were divided into three experimental groups based on the type of dental implants and peri-implant bone grafting as following:

1. Solid Dental Implants (SI, *n*1 = 12): Tapered Screw-Vent^®^, Zimmer Dental Inc., Carlsbad, CA, USA.
2. Porous Dental Implants (PI, *n*2 = 12): Trabecular Metal^®^, Zimmer Dental Inc., Carlsbad, CA, USA.
3. Porous Dental Implants with BCP peri-implant bone grafting (PI + BCP, *n*3 = 12): Trabecular Metal^®^ Implant placement was combined with peri-implant bone grafting using biphasic calcium phosphate (BCP) granulates (Maxresorb^®^, Biotiss Biomaterials, Zossen, Germany). Maxresorb^®^ granules, composed of 60% hydroxyapatite (HA) and 40% beta-tricalcium phosphate (β-TCP).

### 2.3. Sample Size Calculation

The sample number estimation was calculated based on a power analysis using the following formula: *n*1 = *n*2 = *n*3 = 1 + 2C(s/d)^2^. We assumed a standard deviation (s) of 12.5 and an effect size (d) of 15. C-value was fixed at 7.85 (resulting from 1-β = 0.8 and α = 0.05). According to these assumptions, 6 animals were included. Each goat received 6 implants in the bilateral iliac crests (considered independent). The observation period was 7 weeks to evaluate the peri-implant bone healing around solid implants, and surface-porous implants with and without BCP peri-implant bone grafting, based on biomechanical and histomorphometrical assessment.

### 2.4. Surgical Protocol

**Figure 1:**
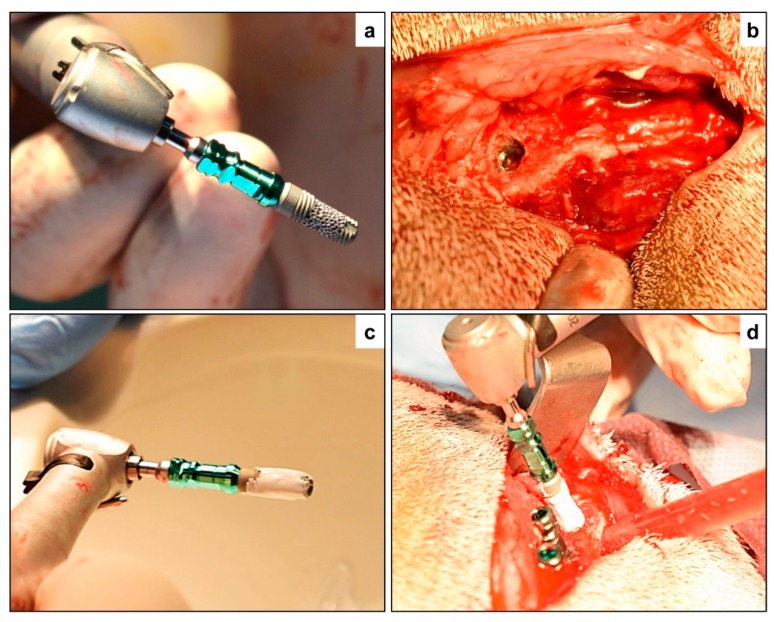
Surgical implant placement—(**a**) Porous titanium implant (PTI) without biphasic calcium phosphate (BCP) augmentation prior to placement, and (**b**) after placement in the iliac crest of the study animal. (**c**) Porous titanium implant augmented with biphasic calcium phosphate (PTI + BCP), and then (**d**) inserted in the iliac crest along with the STI and PTI implants placed in position.

All the surgical procedures were done under general anesthesia (GA) using intramuscular (IM) injection of ketamine hydrochloride (5 mg/kg) and diazepam (1 mg/kg), following strict aseptic protocol. Anesthetic doses were titrated based on the weight of the animal to eliminate any overdose and adverse drug reactions. Following the administration of GA, the animals were immobilized in a ventral position and the bilateral pelvic areas were shaved and disinfected using 7.5% povidone-iodine solution. Following identification and marking of anatomical structures, a transverse skin incision was made starting from the upper medial side of the iliac crest and continuing towards the anterior superior iliac spine laterally on both sides of the vertebral column. The incision through the soft tissues was deepened and the periosteum was detached and elevated to expose the bilateral iliac crest. The drilling of the osteotomy site was done according to the manufacturer’s instructions. Sequential drilling using a slow-speed (800 RPM) contra-angled rotary handpiece along with copious irrigation was carried out until the desired dimensions were achieved depending on the selected implant. Before implant placement, the peri-implant bone grafting with the porous implants (PI) was performed as follows: First, Maxresorb^®^ BCP granules were manually ground to a fine powder and then mixed with sterile saline to enable easy carrying and packing of bone grafting materials. Second, a sterile plastic spatula was used to pack bone materials around and inside the porous structure of PI implants before placement, as shown in Figure 1. By doing this, we made sure that a portion of BCP fine granules lies within the porous structure of the PI implant, and the remainder surrounds the implants. To standardize the bone graft procedure, the same volume (~0.15 cm^3^) of the BCP fine granules was packed around and inside the porous structure of the PI implants by the controlled force allowing even distribution of the grafted materials.

For the implant placement procedures, we prepared the osteotomy width at the coronal aspects to minimize friction between the bone and PI implant surface. Implants were inserted in the osteotomy site at a speed of 20–30 rpm using a specific implant driver. During implant placement, care was taken to not displace BCP materials away from the surface of the PI implant during insertion. One implant from each group was placed on every iliac crest, with an inter-implant distance of 3–4 mm. After insertion of the implants, cover screws were placed on the implants to reduce soft tissue interposition. The sub-cutaneous soft tissues and skin were then closed in layers with Vicryl 2–0 (Polyglactin 910, ETHICON Inc., Bridgewater, NJ, USA) resorbable sutures. To reduce the risk of infection, all animals were administered antibiotics pre-operatively (10 mg/kg intravenously; Amoxicillin^®^, Wockhardt UK Ltd., Uxbridge, UK) and postoperatively, at day 1 and day 3 (50 mg/kg intramuscularly; Albipen^®^, Wockhardt UK Ltd., Uxbridge, UK). To alleviate pain, analgesic (1 mg/kg interamuscularly; Finadyne^®^, Wockhardt UK Ltd., Uxbridge, UK) was administered every 8 h for 2 days. Animals were housed together in a goat farm under veterinary supervision.

### 2.5. Study Endpoint and Specimen Collection

After 7 weeks of follow up, all goats were euthanized with an overdose of Nembutal^®^ 200 mg/mL (Apharmo, Arnhem, The Netherlands). Following euthanasia, the bilateral iliac crests were harvested and all surrounding soft tissues were removed. The region of interest, namely the implant bearing region of the iliac crest, was separated using a diamond blade saw and the exposed cover screws were removed. The iliac crest specimens were then divided equally, and 6 specimens were stored on ice and transported for biomechanical assessment. The remaining 6 iliac crest specimens were divided into smaller blocks of bone surrounding each individual implant and fixed (one week) in a 10% formalin solution for histomorphometrical assessment.

## 3. Analytical Protocols

### 3.1. Biomechanical Testing

**Figure 2:**
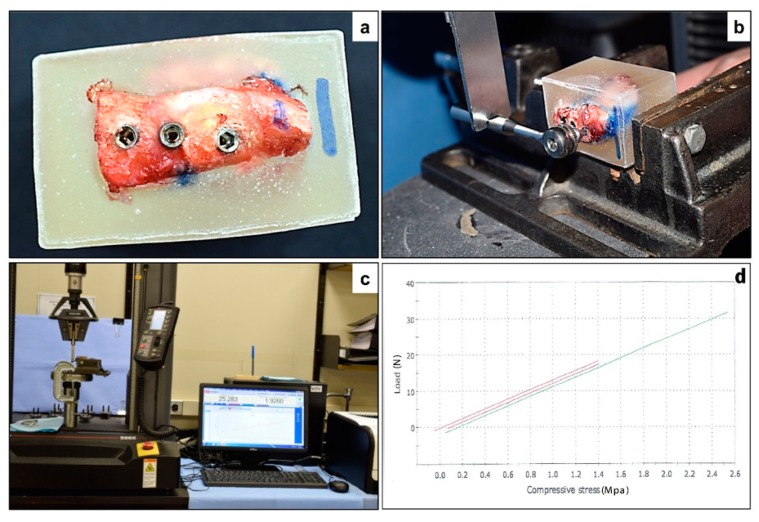
Biomechanical testing of the implants—(**a**) Iliac crest specimen embedded in an acrylic mold, with exposure of the implant bearing surface. (**b**) The INSTRON instrument was used to apply downward force on a lever arm (hand wrench) attached to the implant through a suitable coupling (hexagonal drivers). (**c**) The force was carried out with a load cell of 5 kN and a speed of 5 mm/min. (**d**) Maximum load (N) was selected to present the results of mechanical testing as for the three types of implants.

The specimens segregated for biomechanical assessment were freshly transported to the laboratory and embedded in a mold filled with self-polymerizing acrylic resin. The acrylic blocks were trimmed and fixed to a mechanical testing machine (INSTRON, Instron Corporation, Norwood, MA, USA). The INSTRON instrument was used to apply downward force on a lever arm (hand wrench) attached to the implant through a suitable coupling (hexagonal drivers; Figure 2). The force was carried out with a load cell of 5 kN and a speed of 5 mm/min. The maximum load necessary to advance the lever arm (attached to the implant) downward was defined as the maximum force (N).

### 3.2. Histological and Histomorphometrical Evaluation

**Figure 3:**
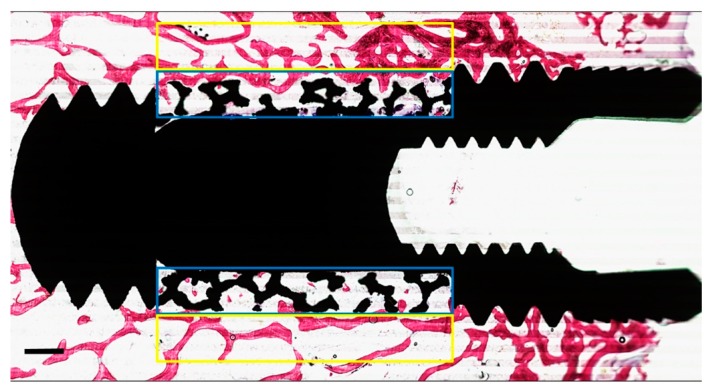
Representative of the two regions of interest (ROI) on either side of the implant. The “yellow box” corresponds to the area outside the porous structure used to measure the peri-implant bone area (PIBA%). The “blue box” corresponds to the area inside the porous structure used to measure bone growth area (BGA%). The mean values obtained from the two ROI were calculated per implant. (Scale bars = 0.5 mm).

The formalin-fixed specimens segregated for histological assessment were dehydrated in a graded series of ethanol (70–100%), washed with acetone, and embedded (non-decalcified) into poly (methyl methacrylate) (pMMA) resin. After polymerization of the pMMA, three sections (parallel to the long axis of the implant) were prepared from each specimen. The thickness of each cutting was ~10 μm and done with a modified diamond blade sawing microtome technique as described previously [31]. The sections were stained using methylene blue and basic fuchsin. The primary evaluation of bone healing around the implant was analyzed by histological as well as histomorphometrical methods using a light microscope (Aperio Technologies, Vista, CA, USA). ImageJ^®^ analysis software (ImageJ v.1.3.8, National Institutes of Health, Bethesda, MD, USA) was used for histomorphometrical evaluation. In addition, quantitative assessment of the percentage of peri-implant bone area (BA%) was measured within a rectangular region of interest (ROI) with a length of 5 mm equal to the porous portion of the implant and a height of 0.5 mm (adjacent to the implant surface). New bone formation within the porous microstructure of each histological section in the PI and the PI + BCP groups was also measured by calculating the percentage of bone growth area (BGA%) within another rectangular ROI (5 mm in length, and 0.5 mm in height) as the overlapping the porous portion of implant (Figure 3).

### 3.3. Statistical Analysis

All quantitative data (maximum force (N), BA%, and BGA%) were expressed as a mean ± SD. One-way analysis of variance (ANOVA) with a Tukey–Kramer multiple comparisons post-hoc test was conducted to compare the difference between the three groups regarding the mechanical test and PIBA%. For BGA%, Student’s T-test was used to compare the means of PTI and PTI + BCP groups. All statistical calculations were performed using Instat software version 3.05, (GraphPad Software Inc., San Diego, CA, USA). Differences were considered statistically significant when *p* < 0.05.

## 4. Results

### 4.1. Post-Operative Observations

A total of 36 implants (*n* = 12 implants per group) were placed in the six study animals. The post-operative healing in all the study animals was uneventful and no morbidities or mortalities were reported until the end of the study period.

### 4.2. Biomechanical Testing (n = 6 Implants Per Group)

The biomechanical evaluation revealed no significant differences in the mean values between the three experimental groups: SI group (19.05 ± 10.19 N), PI group (25.69 ± 4.31 N), and PI + BCP group (18.57 ± 7.57 N) (Table 1).

### 4.3. Histologic and Histomorphometrical Analysis (n = 6 Implants Per Group)

**Figure 4:**
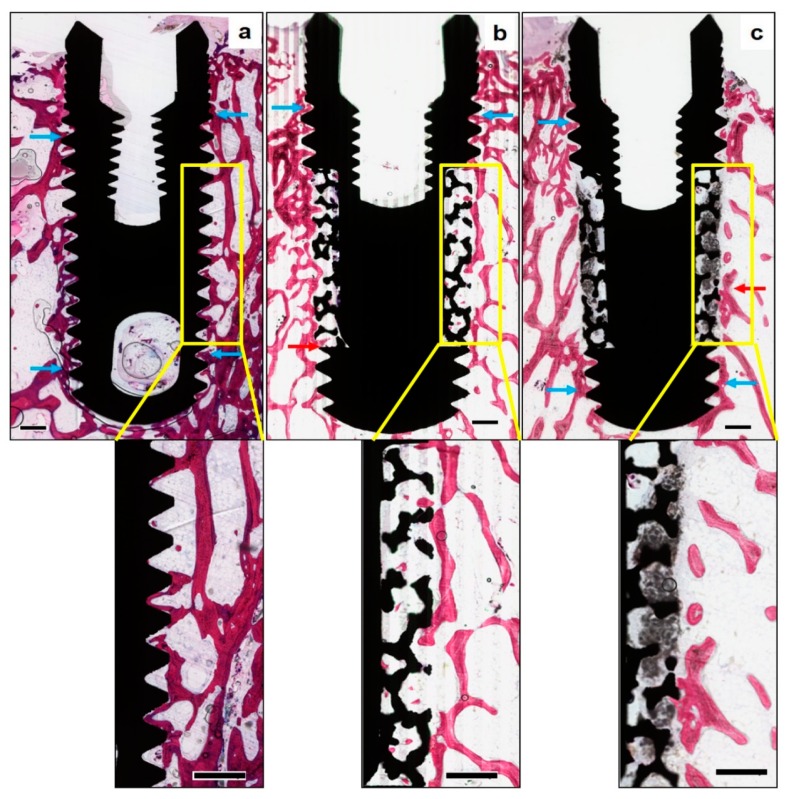
Histomorphometrical assessment of bone formation around the implants—(Methylene blue—basic fuchsin stain, original magnification × 10)—Representative non-decalcified histological sections of the three study groups, (**a**) solid titanium implant (STI), (**b**) porous titanium implant (PTI), and (**c**) porous titanium implant with biphasic calcium phosphate (PTI + BCP), revealing new bone formation around all the implant surfaces along with certain areas of intimate bone to implant contact (blue arrows). Bone ingrowth in the PTI implant (red arrow) is seen up to the inner aspect of the porous structure. BCP granules are seen filling the entire thickness of the porous structure in the PTI+BCP implant, along with evidence of new bone formation and resorption of BCP only in the peripheries (red arrow) and without any signs of BCP resorption in the inner aspect. The “Yellow box” inset is the region of interest (original magnification × 25), measuring 5 mm long by 1000 µm in height, along with the middle third of the implant surfaces. (Scale bars = 0.5 mm).

In Figure 4, qualitative histological observations revealed newly formed bone surrounding the implants in all the study groups. While bone formation was observed around all the examined implant surfaces, certain areas revealed an intimate contact between the implant surface and newly formed bone, along with the ingrowth of new bone within the surface porosities of PI implants. In the area of porous structures, the trabeculae were sparse, irregular, and disconnected with old bone at the periphery. In contrast, no bone ingrowth into the porous portion was observed in the PI + BCP implants. At higher magnifications, the BCP granules were found to fill well the porous structures of the implants, in addition to penetrating into the entire porous zone. While the periphery of the porous zone exhibited evidence of BCP resorption, the core was still filled with BCP granules.

Quantitatively, no statistically significant differences were observed between the three experimental groups with respect to their mean BA% values as presented in Table 1. However, the mean values for percentage of bone growth area (BGA%) were higher (*p* < 0.05) for PI (10.67 ± 4.61%) compared to PI + BCP (6.50 ± 6.53%) (Table 1).

## 5. Discussion

In the current study, our aim was to analyze bone formation around and inside the surface porosity of dental implants with and without the use of peri-implant bone grafting (biphasic calcium phosphate; BCP). We hypothesized that BCP within the surface-porous implants would act as an osteoconductive substrate, and hence accelerate the rate of early bone formation and extent of its ingrowth in relation to surface-porous implants. Our data demonstrated that the use of peri-implant BCP grafting in combination with surface-porous implants exhibited less new bone ingrowth in the early stage of healing compared to surface-porous implants without BCP grafting. Assessment of the improved peri-implant bone healing of surface-porous implants over solid implants was also done and showed no significant differences. Furthermore, after 7 weeks of healing in the iliac crest of a goat model, all implants demonstrated comparable mechanical strength with no significant differences among the groups.

Undoubtedly, the biocompatibility of titanium has encouraged its utilization for dental implant manufacturing over the years [33]. Different methods have been developed to increase the surface bioactivity and topography of dental implants [34,35]. Nowadays, immense efforts by researchers, using several new technologies, are applied to improve titanium implant surface and topography to promote an early process of osseointegration [35].

Although its osteoconductivity seems limited, surface modifications in surface-porous titanium implants have greatly improved their biological properties [7,17]. It is claimed that the surface properties of porous implants directly influence osteogenesis at the bone–implant interface, owing to the enhanced biological anchorage and a greater contact area [5,7]. The porous implants used in the present study were Trabecular-Metal^®^ Implants designed with a pore size of up to 500 µm, which reportedly promote bone ingrowth [36,37]. The porous portion occupies approximately 40% by volume of the total body of the implant [36]. This is advantageous as a result of increasing the bone–implant interface area by at least three times compared to that of a conventional solid implant [36]. Additionally, the possibility of bone ingrowth within the trabecular structures of porous implants might enhance their mechanical and biological properties [37].

Although the present histomorphometrical analysis confirmed new bone growth within the porous structures of Trabecular-Metal^®^ implants, the mechanical assessment revealed no significant differences between the three types of implants. This is in concurrence with the findings reported in two earlier preclinical studies [38,39], in which new bone growth within the porous structure of implants, at 6 weeks, was limited and showed no improved biomechanical function. However, Bobyn et al. [40] tested implants with a pore size of approximately 50 to 400 µm and reported an increase in biomechanical values following an 8-week healing period. While the increased pore size was associated with increasing strength of fixation, as reported in several studies [41,42,43,44], some other studies claim that the amount of new bone ingrowth and strength of fixation are independent of the pore sizes [45,46]. Therefore, prospective pre-clinical trials would still be needed to examine the assumption that surface-porous dental implants (with different pore size) are really superior to conventional dental implants.

In clinical scenarios impeded by insufficient alveolar bone mandating bone augmentation, surface-porous implants could serve as a potential alternative for simultaneous implant placement with bone augmentation (e.g., sinus augmentation, large dental-extraction socket) [47,48]. Owing to the paucity of evidence related to this topic, the present study also evaluated the effect of supplemental CaP-bone substitute within surface-porous implants. It was indeed hypothesized that BCP granules would enhance bone-implant ingrowth in the goat iliac crest model, which is analogous to low-quality bone in clinical practice [49]. The concept of peri-implant bone grafting with CaP-based materials has been applied in orthopedics to improve pedicle screw fixation in patients with poor bone quality [50,51,52]. Interestingly, there was no histological evidence of bone ingrowth into the porous structures of implants placed with peri-implant BCP grafting after 7 weeks of healing. While the BCP granules were observed over the entire porous zone, their effect on bone formation was evidenced only at the periphery and the core remained largely unaltered. Hence, it is tempting to assume that BCP granulate did not resorb as fast as was expected and failed to form an interconnecting porosity for new bone deposition.

Bioactive ceramics such as BCP have been proven to be a promising bone substitute for bone regeneration [53,54] and previous animal studies have reported new bone ingrowth until the core of the osseous defects when augmented with BCP [55,56,57,58]. These studies concluded that BCP granules promote bone ingrowth through osteoconduction. The bioactivity of BCP is mainly related to the resorption of the β-TCP particles and subsequent bone formation and binding to the HA particles. However, the rates of BCP degradation and bone ingrowth do not always match and could restrain the biological properties of bone ceramics [58]. The results of the present study can be attributed to several limitations including the short evaluation period and the method of applying BCP granules on PI implants. In this experiment, Maxresorb^®^ BCP granules were manually ground to a fine powder and then mixed with sterile saline to enable the easy application of the BCP materials into the porous surface of the implants. Healing time is also recommended to be more than 7 weeks for progressive resorption of BCP granules and coordinated bone formation within the porous structure of implants [58].

Among the various animal models available for assessment of the osteogenic performance of newly developed implant surfaces, the present study adopted the validated goat iliac crest model, although not regarded as a load-bearing bone model [32]. In addition, evidence-based research considers the iliac crest model analogous to human bone in composition and as a translational model mimicking low-quality bone [32].

## 6. Conclusions

The present study showed that peri-implant bone grafting of the surface-porous implant with synthetic BCP does not significantly accelerate early osseointegration bone ingrowth. Assessment of peri-implant bone healing around solid implants, porous implants and porous implants with BCP grafting were comparable at 7 weeks.

## Figures and Tables

**Table 1:** Mean ± SD values of biomechanical and histomorphometrical quantitative variables in the three experimental groups.

Quantitative Variables	STI Implants		PTI Implants		PTI + BCP Implants		-
	N	Mean ± SD	N	Mean ± SD	N	Mean ± SD	
Maximum load (N)	6	19.05 ± 10.19	6	25.69 ± 4.31	6	18.57 ± 7.57	*p* > 0.05 *
Peri-implant Bone Area (PIBA%)	6	8.63 ± 3.93	6	9.89 ± 3.69	6	9.28 ± 2.61	*p* > 0.05 *
Bone growth Area (BGA%)		-		10.67 ± 4.61		6.50 ± 6.53	*p* < 0.05

* There is no statistical significance based on the ANOVA test. There is statistical significance based on the Student’s *T*-test.
